# A tightly regulated IL-22 response maintains immune functions and homeostasis in systemic viral infection

**DOI:** 10.1038/s41598-017-04260-0

**Published:** 2017-06-20

**Authors:** Panpan Yi, Yuejin Liang, Denley Ming Kee Yuan, Zuliang Jie, Zakari Kwota, Yan Chen, Yingzi Cong, Xuegong Fan, Jiaren Sun

**Affiliations:** 1 Department of Infectious Diseases, Key Laboratory of Viral Hepatitis of Hunan, Xiangya Hospital, Central South University, Hunan, China; 2 Department of Microbiology and Immunology, University of Texas Medical Branch, Texas, USA; 3 Department of Ophthalmology, University of Texas Medical Branch, Texas, USA; 4 Department of Pathology, University of Texas Medical Branch, Texas, USA

## Abstract

Interleukin-22 (IL-22) plays an important role in host immunity and tissue homeostasis in infectious and inflammatory diseases. However, the function and regulation of IL-22 in viral infection remain largely unknown. Here, we report that viral infection triggered early IL-22 production from the liver and lymphoid organs. γδ T cells are the main immune cells to produce IL-22 in the liver, a process mediated by the IL-23/phosphoinositide 3-kinase (PI3K)/mammalian target of rapamycin complex 1 (mTORC1) signaling pathway. In the presence of IL-23, IL-22 production is independent of aryl hydrocarbon receptor (AhR) signaling. In acute and persistent viral infections, IL-22 deficiency resulted in thymic and splenic hypertrophy, while excessive IL-22 induced atrophy in these lymphoid organs. Moreover, IL-22 deficiency enhanced T cell responses to promote viral clearance, but increased IL-22 *in vivo* decreased T cell numbers and functions in the liver and lymphoid tissues. Together, our findings reveal a significant effect of the IL-23/PI3K/mTORC1 axis on regulating IL-22 production and also identify a novel role of IL-22 in controlling antiviral T cell responses in the non-lymphoid and lymphoid organs during acute and persistent viral infections.

## Introduction

Interleukin-22 (IL-22) has been linked to a number of inflammatory conditions, including inflammatory liver diseases, inflammatory gut diseases, and systemic inflammation^1, 2^. IL-22 plays a major role in tissue regeneration and host defense against microbes at barrier surfaces^1–4^. Although the role of IL-22 in bacterial and fungal infections is well-defined, the sources of IL-22, regulatory mechanisms of its production, as well as its function in acute and chronic viral infections remain elusive. The regulation of IL-22 production is dependent on the milieu stimuli and transcriptional factors in many inflammatory disorders^1, 5–8^. IL-23 has been reported to be associated with IL-22 expression by nature killer (NK) T cells upon influenza exposure^9^. However, little is known about its down-stream signaling pathway in regulating IL-22 production. Recently, the phosphoinositol-3-kinase (PI3K)/mammalian target of rapamycin complex 1 (mTORC1) signaling pathway has been considered crucial for mediating T cell differentiation^10, 11^. However, it is unclear whether the PI3K/mTORC1 signaling pathway is involved in modulating IL-23-induced IL-22 production in viral infection.

The antiviral activity of IL-22 has been implied in rotavirus infection^12–14^. IL-22 is up-regulated in patients with chronic hepatitis B virus (HBV) and hepatitis C virus (HCV) infections^15–17^. It is also reported to have a pathological role in an HBV transgenic mouse model and to induce an acute-phase response in systemic physiology^16, 18^, indicating it is a possible contributory factor in viral pathogenesis in certain contexts^14^. To date, whether IL-22 up-regulation in these distinct conditions is protective or pro-inflammatory is not clear; therefore, it is imperative to further define the mechanistic actions of IL-22 in viral infections.

In this study, we infected mice with lymphocytic choriomeningitis virus (LCMV). Viral infection triggered IL-22 production from liver, spleen and thymus tissues. γδ T cells were the main subtype of immune cells to produce IL-22 in the liver, a process that is regulated by the IL-23/PI3K/mTORC1 signaling pathway, rather than by traditional aryl hydrocarbon receptor (AhR) signaling. Importantly, we found that IL-22 was crucial to restrict effector T cell responses, and contributed to the impediment of viral elimination in the liver and lymphoid organs during acute and persistent viral infections. In addition, IL-22 deficit resulted in hypertrophy in the spleen and thymus, while over-expression of IL-22 in viral infections induced splenic and thymic atrophy, which most likely is a contributory mechanism for IL-22 to suppress T cell responses. Thus, our data suggest that LCMV infection elicits IL-22 expression from innate immune cells through the IL-23/PI3K/mTOR axis, and its production is essential for modulating antiviral T cell responses in both non-lymphoid and lymphoid tissues during acute and persistent viral infections.

## Results

### Viral infection elicits early IL-22 production from γδ T cells

**Figure 1:**
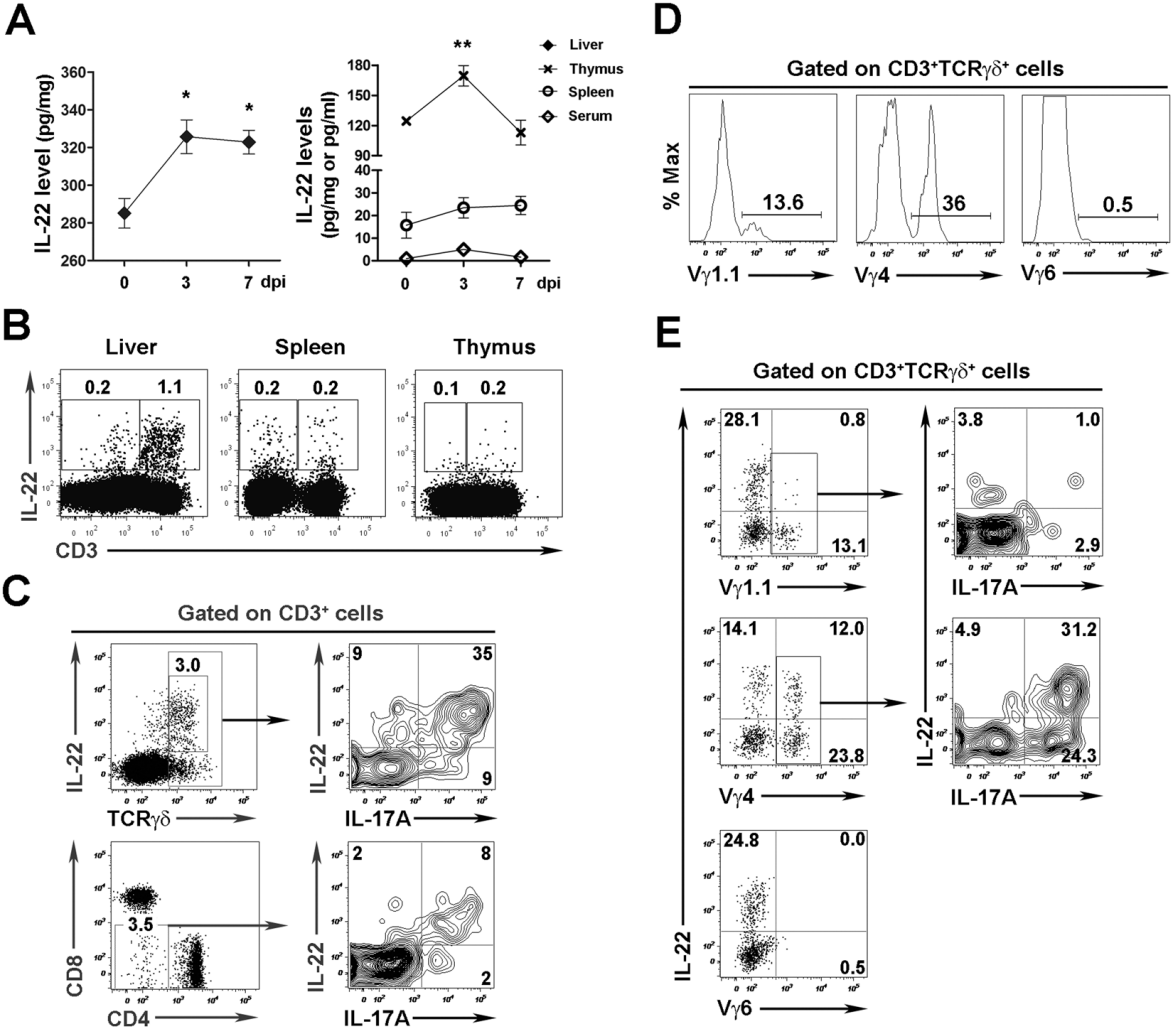
Viral infection triggers IL-22 production in γδ T cells. C57BL/6 (B6) mice were *i*.*v*. injected with LCMV Clone 13 (2 × 10^6^ pfu/mouse) and then sacrificed at 0, 3, 7 dpi. (**A**) IL-22 levels in the liver, thymus, spleen, and serum were analyzed via ELISA. The results are expressed as the mean ± SEM (*n* = 3–4 mice per time point). *p < 0.05; **p < 0.01. (**B** to **E**) Introhepatic lymphocytes (IHLs) were isolated at 3 dpi and stimulated with rIL-23 (20 ng/ml) overnight. (**B**) IL-22 expression in CD3^+^ and CD3^−^ cells in the liver, spleen and thymus were analyzed by flow cytometry. (**C**) Representative plots of IL-22 and IL-17 expression in hepatic γδ T cells and DNT cells. Percentage of subsets of γδ T cells in the liver (**D**), and IL-22 and IL-17 expression in the subtypes (**E**) were analyzed by flow cytometry. Data are representative of three independent experiments.

To determine the dynamic expression pattern of IL-22 in viral infection, we *i*.*v*. infected C57BL/6 (B6) mice with LCMV Clone 13. We found that IL-22 protein level in the liver reached a peak at 3 days post infection (dpi) (Fig. 1A). IL-22 production in the thymus also reached a maximum level at 3 dpi and declined at 7 dpi. Additionally, IL-22 levels were very low in the spleen and serum (Fig. 1A).

To identify the source of IL-22, we analyzed the IL-22-expressing cells in various organs and found that most IL-22^+^ cells were CD3^+^ T cells, especially those in the liver (Fig. 1B). We further found that γδ T cells prominently expressed IL-22 as well as IL-17A in the liver after viral infection (Fig. 1C). In addition to γδ T cells, double-negative T cells (DNT, CD3^+^CD4^−^CD8^−^NK1.1^−^TCRγδ^−^) also produced IL-22, although to a less extent (Fig. 1C, Supplementary Fig. S1). Myeloid cells such as monocytes/macrophages, neutrophils, and DCs produced negligible levels of IL-22 in viral infection (Supplementary Fig. S1). Although innate lymphoid cells (ILCs) were a significant source of IL-17 production in the liver, few expressed IL-22 (Supplementary Fig. S1C). Moreover, CD4^+^ cells, which are either T cells or lymphoid tissue inducer (LTi) cells^19^, expressed very low IL-22 (Supplementary Fig. S1A and D). Our data suggested that hepatic ILCs were not a dominant producer of IL-22 in LCMV infection. Thus, γδ T cells appeared to be the main producers of IL-22 in mice exposed to viruses. Consistent with previous studies^20, 21^, most hepatic γδ T cells were Vγ4^+^ and Vγ1.1^+^, rather than Vγ6^+^ (Fig. 1D). Furthermore, we found that Vγ4^+^γδ T cells, but not the Vγ1.1^+^ or Vγ6^+^ γδ T cells, were the main subtype in LCMV infection that produced IL-22 in the liver (Fig. 1E).

### PI3K/mTOR pathway regulates IL-22 expression from γδ T cells in viral infection

**Figure 2:**
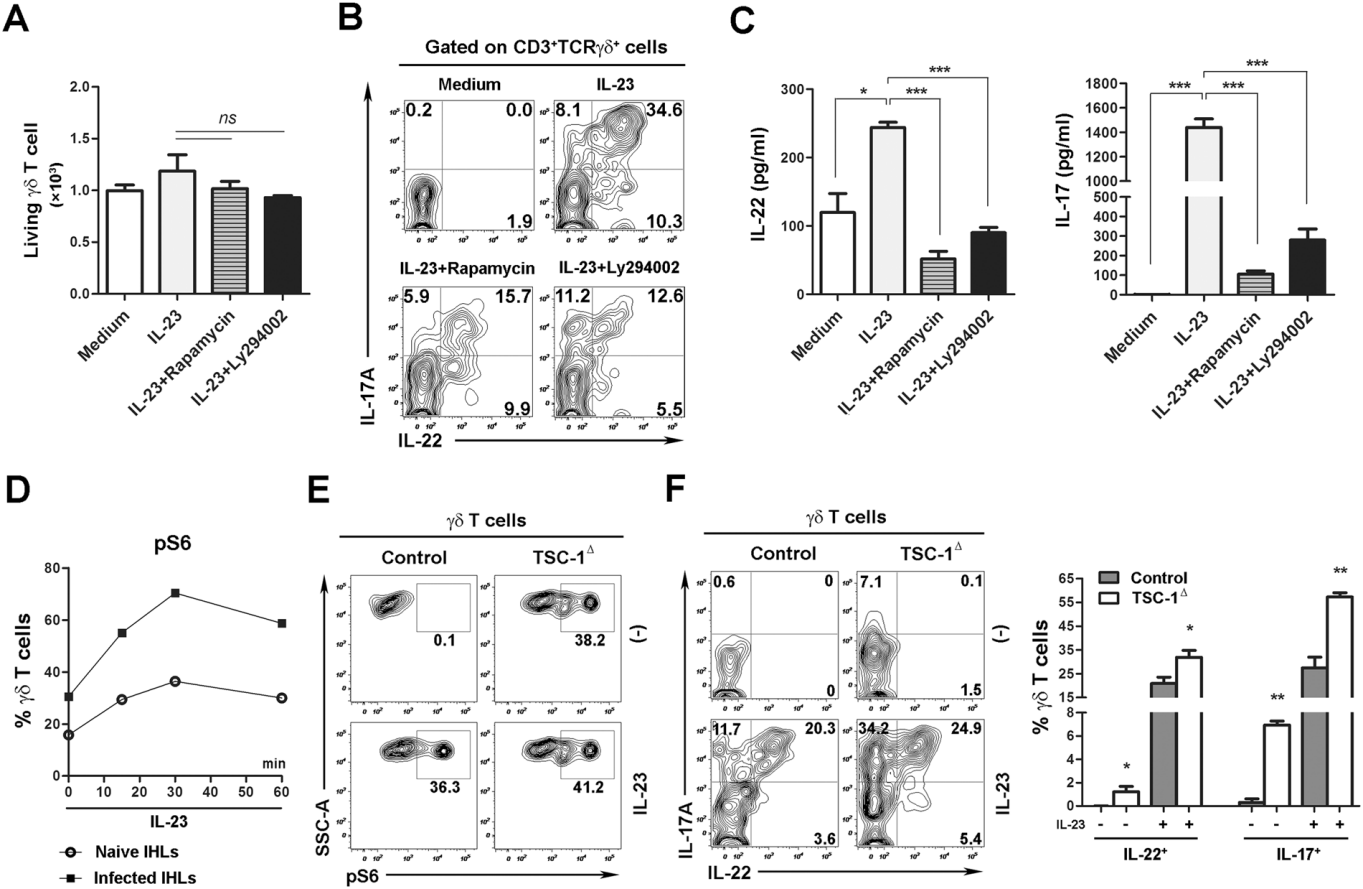
PI3K/mTOR pathway regulates IL-22 and IL-17 expression in virus-exposed γδ T cells. (**A** to **D**) IHLs were isolated from Clone 13-infected B6 mice at 3 dpi and then cultured with indicated conditions overnight. IL-23 (20 ng/ml); Rapamycin (25 nM), mTOR inhibitor; Ly294002 (5 μM), PI3K inhibitor. (**A**) The living γδ T cells were stained with live dye and enumerated using flow cytometry. (**B**) IL-22 and IL-17A expression in γδ T cells was analyzed by flow cytometry. (**C**) IHLs were incubated with indicated conditions *in vitro* for 3 days, and then IL-22 and IL-17 levels in the supernatants were determined by ELISA. (**D**) IHLs of naïve or infected B6 mice were treated with IL-23 as indicated. Flow cytometry was used to detect pS6 expression in γδ T cells. (**E** and **F**) TSC-1-deficient (TSC-1^Δ^) or control mice were challenged with Clone 13 (2 × 10^6^ pfu/mouse). IHLs were isolated at 3 dpi. (**E**) IHLs were cultured with or without IL-23 for 30 min. Expression of pS6 was examined by flow cytometry. (**F**) IHLs were cultured with or without IL-23 overnight. IL-22 and IL-17A expression in γδ T cells was detected by flow cytometry. Data are representative of three independent experiments. The results are expressed as the mean ± SEM (*n* = 3–5). *p < 0.05; **p < 0.01; ***p < 0.001.

The mammalian target of rapamycin (mTOR) is a serine/threonine protein kinase that belongs to the PI3K-related protein kinase family and regulates cell growth, proliferation, and differentiation^10, 11, 22^. IL-23 is responsible for activating the PI3K/Akt pathway to promote IL-17 expression in CD4^+^ T cells^23^. However, it remains unclear as to whether the signaling pathway regulates IL-22 production in viral infection. To address this question, we isolated intrahepatic lymphocytes (IHLs) from LCMV-infected mice at 3 dpi and treated these cells *in vitro* with IL-23, in the presence or absence of PI3K inhibitor (Ly294002) or mTOR complex 1 (mTORC1) inhibitor (rapamycin). No significant toxicity was observed by the treatments of these inhibitors in the indicated concentrations *in vitro* (Fig. 2A). IL-23 treatment significantly promoted both IL-22 and IL-17A expression in γδ T cells. Rapamycin and Ly294002 dramatically suppressed the stimulatory effects of IL-23 on IL-22 and IL-17A production (Fig. 2B). IL-22 as well as IL-17 levels in the supernatant were suppressed consistently by rapamycin and Ly294002 (Fig. 2C).

To further investigate the mechanistic action of the PI3K/mTOR signaling pathway in viral infection, we measured mTORC1 activation by monitoring the phosphorylation status of ribosomal protein S6 (pS6), a down-stream target of the mTORC1-mediated pathway^24^. Hepatic γδ T cells of virus-infected mice had a higher level of pS6 (30% positive) compared to that in the naïve cells (18% positive) (Fig. 2D). IL-23 treatment *in vitro* greatly increased pS6 levels in virus-exposed hepatic γδ T cells, suggesting an IL-23-mediated activation of the mTORC1 signaling pathway in these cells. To further confirm that the activation of the IL-23/PI3K/mTOR axis affects IL-22 production *in vivo*, we depleted the expression of TSC1 (the up-stream negative regulator of mTORC1) by injecting *TSC1* ^fl/fl^ mice with AAV5-Cre (TSC-1^Δ^) or control vector (AAV5)^25–27^, and then challenged these animals with LCMV. Hepatic γδ T cells from TSC-1^Δ^ mice had a higher level of pS6, which confirms the up-regulated mTORC1 activity (Fig. 2E). Importantly, TSC1-deficient γδ T cells exhibited increased IL-22 and IL-17A expression, and this promotion was augmented by IL-23 (Fig. 2F). Then taken together, these data from LCMV infection studies supported results showing that IL-22 production from γδ T cells was tightly regulated via the IL-23/PI3K/mTOR axis.

### AhR signaling is dispensable for IL-23-induced IL-22 production in γδ T cells

**Figure 3:**
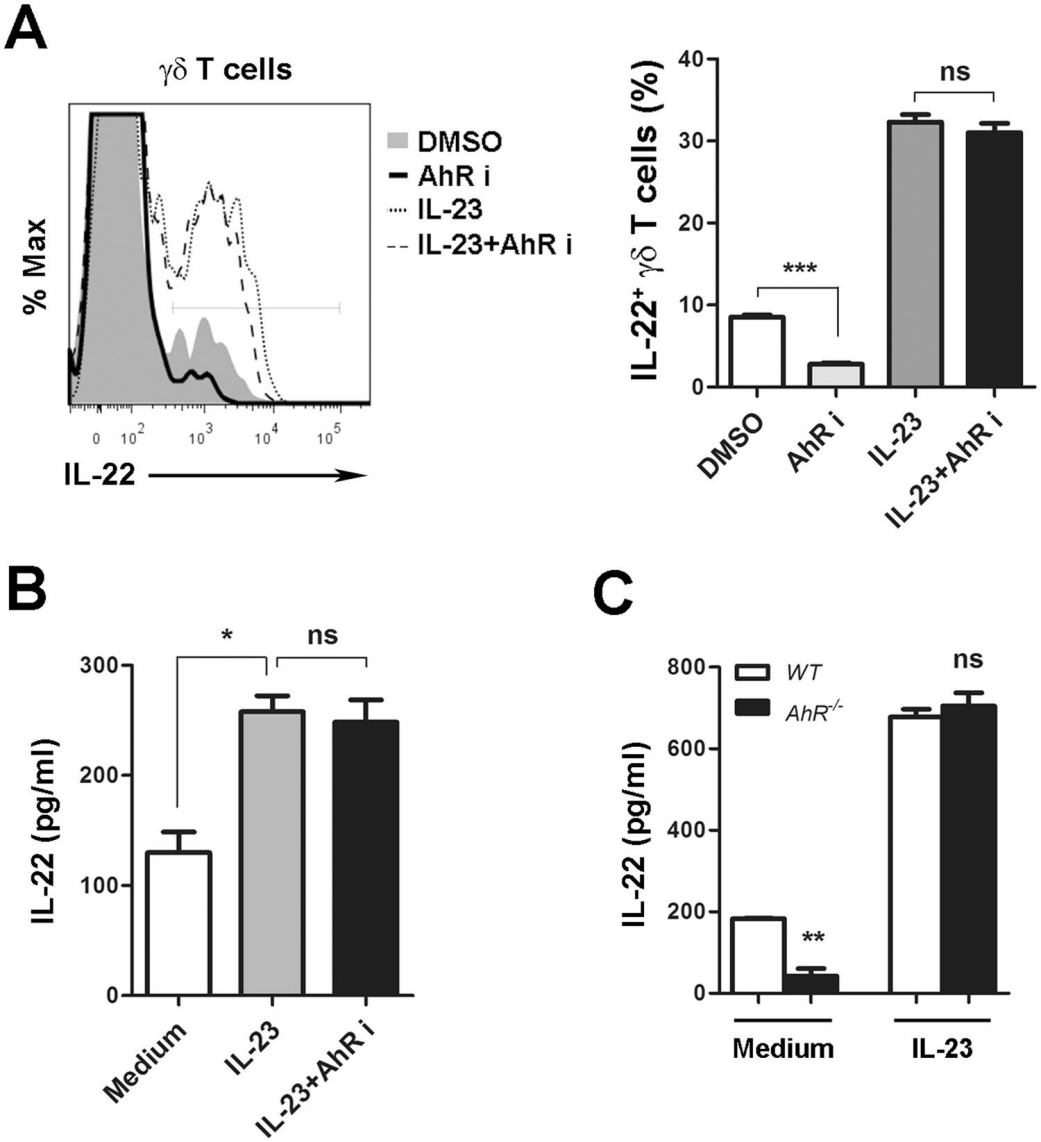
IL-22 expression in γδ T cells is independent of AhR signaling in the presence of IL-23. (**A** and **B**) Clone 13-induced IHLs at 3 dpi were cultured at the indicated conditions overnight. IL-23 (20 ng/ml); AhR i (3 μM), AhR inhibitor. (**A**) The percentage of IL-22-expressing γδ T cells was tested by flow cytometry. (**B**) IHLs were incubated under described conditions *in vitro* for 3 days, and then IL-22 levels in the supernatant were examined with ELISA. (**C**) IHLs from WT and *AhR* ^−/−^ mice were incubated with Clone 13 (1 × 10^6^ pfu/ml) and stimulated with or without IL-23 (20 ng/ml) *in vitro* for 3 days. IL-22 levels in the supernatants were determined by ELISA. Data are representative of two independent experiments. The results are expressed as the mean ± SEM (*n* = 3). *p < 0.05; **p < 0.01; ***p < 0.001. ns, no significant difference.

The transcriptional factor AhR has been considered as a positive inducer of IL-22 in CD4^+^ T cells and ILCs in the gut^28, 29^. To determine whether AhR regulates IL-22 production from γδ T cells in viral infection, we measured the effects of AhR inhibitor on IL-22 expression in hepatic γδ T cells at 3 dpi. AhR inhibitor reduced IL-22 expression in γδ T cells in the absence of exogenous IL-23 (Fig. 3A). However, in the presence of IL-23, the AhR inhibitor did not impede IL-22 production from γδ T cells (Fig. 3A). Similar results were observed when we analyzed the supernatant IL-22 in the culture of IHLs (Fig. 3B). To confirm this finding, we isolated LCMV-exposed IHLs from wild-type (WT) and *AhR* ^−/−^ mice, and then cultured these cells with or without IL-23. AhR deficiency resulted in lower IL-22 production in the absence of IL-23 stimulation; however, IL-23 administration overcame AhR deficiency and restored the IL-22 secretion (Fig. 3C). These data indicated that in LCMV infection IL-23 regulates IL-22 production in an AhR-independent fashion.

### IL-22 deficiency enhances expansion, recruitment and activity of T cells in acute viral infection

**Figure 4:**
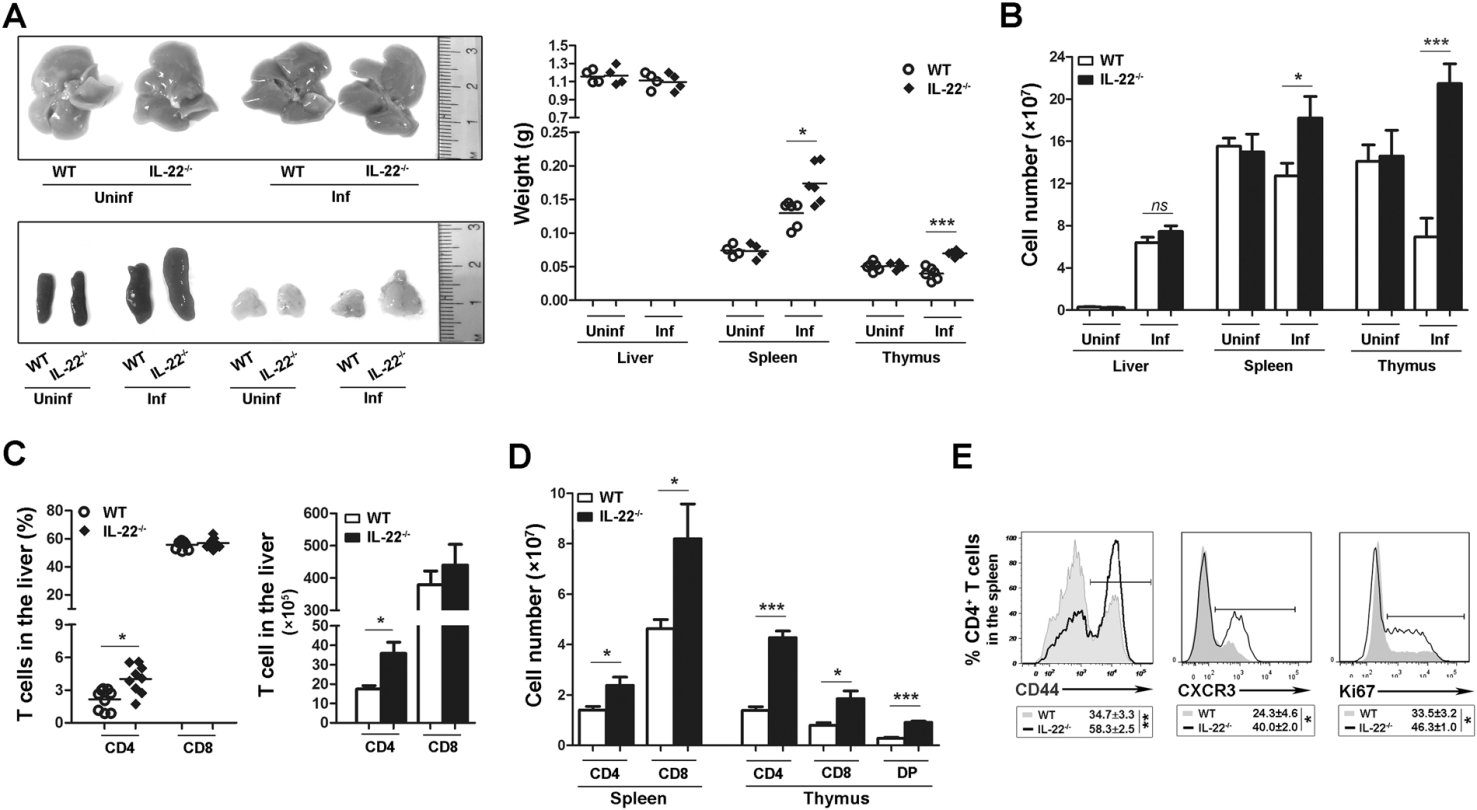
IL-22 deficiency enhances effector T cell expansion in acute viral infection. WT and *IL*-*22* ^−/−^ mice were infected with Arm (2 × 10^5^ pfu/mouse) for 7 days. (**A**) The size and weight of the liver, spleen and thymus were examined. (**B**) The total number of leukocytes in the liver, spleen and thymus were counted. (**C**) Plots for the percentage and number of hepatic CD4^+^ and CD8^+^ T cells. (**D**) Plots for the number of CD4^+^ and CD8^+^ T cells in the spleen and thymus, as well as thymic double-positive (DP) T cells by gating on CD3^+^ cells. (**E**) The expression of CD44, CXCR3, and Ki67 in splenic CD4^+^ T cells was analyzed by flow cytometry. Data are representative of three independent experiments. The results are expressed as the mean ± SEM (*n* = 9). *p < 0.05; **p < 0.01; ***p < 0.001. ns, no significant difference.

To explore whether IL-22 plays a role in acute viral infection, we infected WT and *IL*-*22* ^−/−^ mice with the LCMV Armstrong (Arm) for 7 days. Although a lack of IL-22 had no effect on the size and weight of the liver, it resulted in significant splenomegaly and thymic proliferation (Fig. 4A). In this case, the total number of splenocytes and thymocytes was significantly increased in *IL*-*22* ^−/−^ mice (Fig. 4B). No such difference was observed for the lymphoid organs in non-infected WT and *IL*-*22* ^−/−^ mice (Fig. 4A and B). To identify the role of IL-22 in lymphoid organs, we analyzed IL-22Rα receptor expression in thymic and splenic stromal cells in infected mice. IL-22Rα was expressed in splenic stromal cells (CD45^−^), thymic non-epithelial stromal cells (CD45^−^), and thymic epithelial cells (CD45^−^MHCII^+^), with a frequency of 26%, 9% and 40%, respectively (Supplementary Fig. S2A). These data indicate that in viral infections the IL-22/IL-22R axis is crucial for host responses in lymphoid organs.

We further examined the effects of IL-22 on the output of lymphocytes in acute LCMV infection. CD8^+^ T cells were the dominant lymphocyte population (around 60%) in the livers of WT as well as *IL*-*22* ^−/−^ mice, and the frequency and total number of CD8^+^ T cells in the liver of *IL*-*22* ^−/−^ mice were comparable to those in WT mice (Fig. 4C). In contrast to CD8^+^ T cells, a lack of IL-22 resulted in a doubled frequency and number of CD4^+^ T cells in the liver compared to those found in WT mice (Fig. 4C). In addition to the non-lymphoid organs, IL-22 deficiency also strikingly doubled or even tripled the counts of splenic CD4^+^ or CD8^+^ T cells (Fig. 4D). In the thymus, IL-22 deficiency resulted in increased CD4^+^, CD8^+^ and double-positive (DP) cells when gated on either CD3^+^ cells or total thymocytes (Fig. 4D, Supplementary Fig. S2B and C). We then examined whether IL-22 deficiency impacts the activation, trafficking activities, and proliferation of T cells during acute infections. The absence of IL-22 resulted in an increased expression of the activation marker CD44, chemokine receptor CXCR3, and proliferation marker Ki67 in the splenic CD4^+^ T cells (Fig. 4E). In addition, the elevated T cell output was not a consequence of the cells being refractory to cell death, as IL-22 deficiency did not significantly reduce T cell apoptosis in the thymus and spleen (Supplementary Fig. S2D).

**Figure 5:**
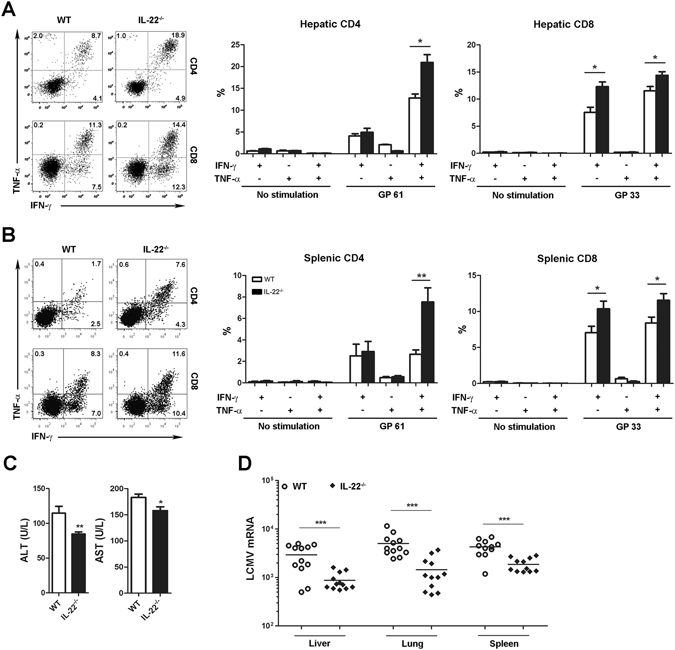
IL-22 deficiency augments T cell anti-viral activities during acute viral infection. WT and *IL*-*22* ^−/−^ mice were infected with LCMV Arm (2 × 10^5^ pfu/mouse) for 7 days. (**A** and **B**) Leukocytes were isolated and incubated with or without LCMV peptide GP 61 or GP 33 for 5 h. IFN-γ and TNF-α production in viral-specific CD4^+^ and CD8^+^ T cells in the liver (**A**) and spleen (**B**) was determined by flow cytometry. (**C**) Serum ALT and AST levels were tested. (**D**) Expression of viral mRNA in the liver, lung and spleen tissues at 7 dpi was determined by qPCR. Data are representative of three independent experiments. The results are expressed as the mean ± SEM (*n* = 9–12). *p < 0.05; **p < 0.01; ***p < 0.001.

Next, we assessed whether a lack of IL-22 affects the function of effector T cells. Lymphocytes in the liver and spleen from LCMV Arm-infected mice at 7 dpi were stimulated with LCMV peptide GP 61 or GP 33 for viral-specific CD4^+^ or CD8^+^ T cells, respectively. IL-22 deficiency resulted in higher frequency of epitope-specific IFN-γ^+^TNF-α^+^ and IFN-γ single-positive CD4^+^ and CD8^+^ T cells in the liver and spleen (Fig. 5A and B). The percentage of TNF-α^+^ population was also increased in IFN-γ^+^CD4^+^ T cells of *IL*-*22* ^−/−^ mice (Supplementary Fig. S2E). Thus, IL-22 affects both polyfunctional and IFN-γ single-positive T cell subtypes. Since few cytokine-producing cells were detected without peptide stimulation (Fig. 5A and B), these data suggest to us that the elevated T cell response is driven by an antigen-specific mechanism rather than by a generalized inflammatory environment. In addition, the absence of IL-22 slightly decreased ALT and AST levels in the serum (Fig. 5C) and slightly attenuated liver histological injuries (Supplementary Fig. S2F). Importantly, intensive viral-specific CD4^+^ and CD8^+^ T cell functions were associated with an obviously decreased viral load in liver, lung and spleen tissues at 7 dpi (Fig. 5D). These data suggest that, during acute LCMV infection, the lack of IL-22 boosts anti-viral T cell responses without significant immunological injuries in inflamed tissues.

### Over-expression of IL-22 shrinks lymphoid organs and dampens T cell response in acute viral infection

**Figure 6:**
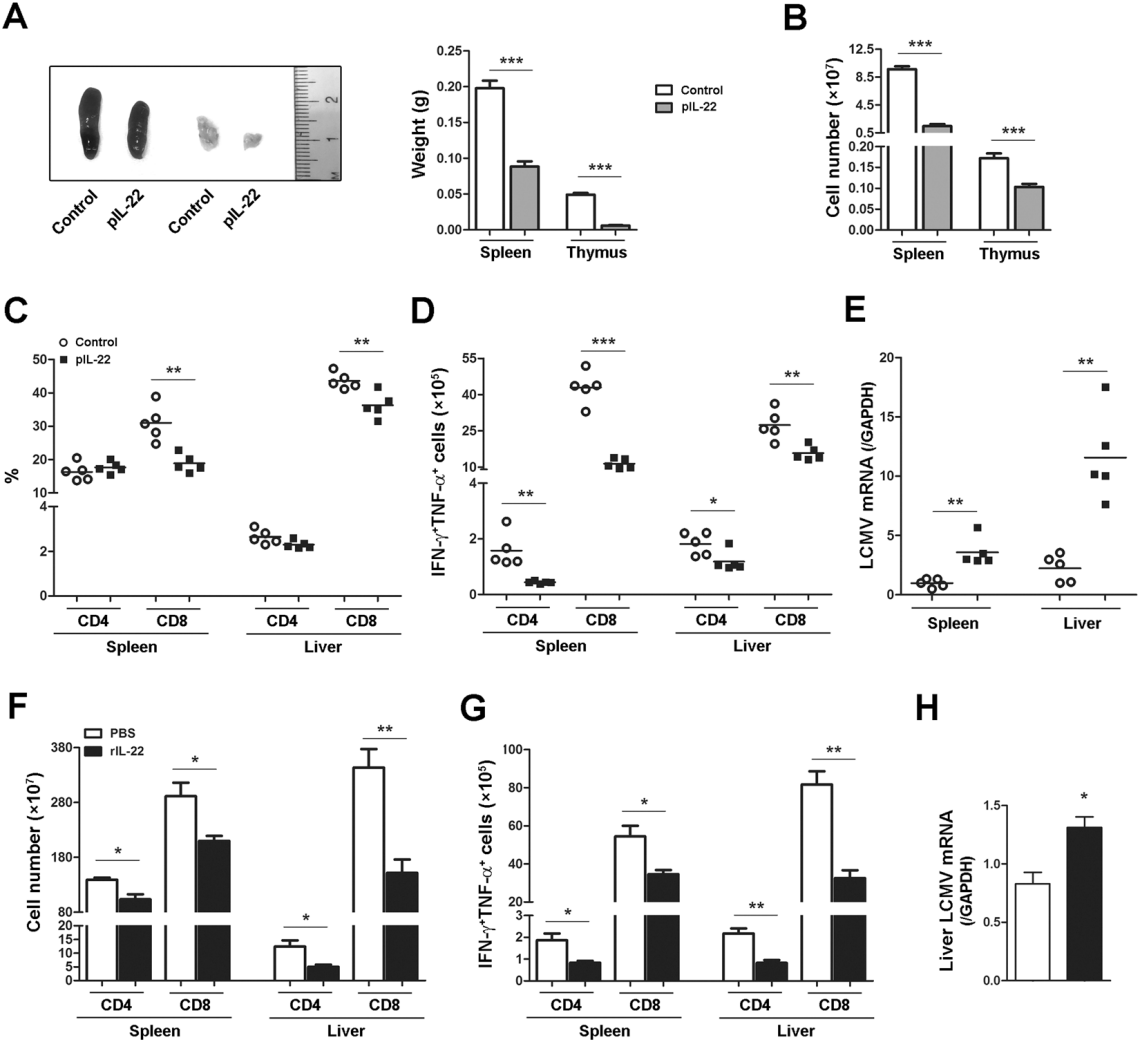
Over-expression of IL-22 dampens effector T cell response in acute viral infection. (**A** to **E**) B6 mice were infected with LCMV Arm (2 × 10^5^ pfu/mouse) at day 0, and then hydrodynamically injected with pRK5-mIL-22 (pIL-22) or pRK5 (control) at 3 and 5 dpi. Mice were sacrificed at 7 dpi. (**A**) The spleen and thymus were weighed. (**B**) The number of leukocytes in the spleen and thymus was counted. (**C**) The percentage of CD4^+^ and CD8^+^ T cells in the spleen and liver were analyzed by flow cytometry. (**D**) The number of IFN-γ^+^TNF-α^+^ CD4 and CD8 T cells in the spleen and liver was determined with flow cytometry. (**E**) Expression of viral mRNA was determined by qPCR. (**F** to **H**) B6 mice were infected with LCMV Arm and then *i*.*p*. injected with recombinant IL-22 protein (5 µg/mouse) or PBS at 1, 3 and 5 dpi. Mice were sacrificed at 7 dpi. (**F**) Plots for the number of splenic or hepatic CD4^+^ and CD8^+^ T cells. (**G**) The number of IFN-γ^+^TNF-α^+^ CD4 and CD8 T cells in the spleen and liver was analyzed by flow cytometry. (**H**) Expression of viral mRNA in the liver was determined by qPCR. Data are representative of two independent experiments. The results are expressed as the mean ± SEM (*n* = 4–5). *p < 0.05; **p < 0.01; ***p < 0.001.

To establish the role of IL-22 in the thymic and splenic pools during acute viral infection, we investigated the effect of IL-22 over-expression on T cell expansion and maintenance in lymphoid pools by the hydrodynamic injection of IL-22 plasmid. IL-22 levels were identified in the thymus, spleen, liver, and lung tissues as well as the serum (Supplementary Fig. S3A). Over-expression of IL-22 resulted in apparent atrophy in the spleen and thymus during acute LCMV infection (Fig. 6A), accompanied by decreased numbers of splenocytes and thymocytes (Fig. 6B). Moreover, IL-22 over-expression decreased the frequency of CD8^+^ T cells in the spleen and liver and also reduced the counts of single-positive T cells, as well as DP cells in the thymus (Fig. 6C, Supplementary Fig. S3B). Excessive IL-22 significantly reduced the number of viral-specific IFN-γ^+^TNF-α^+^ CD4^+^ and CD8^+^ T cells in the spleen and liver (Fig. 6D) and also resulted in a lower proportion of cytokine-producing T cells in the spleen (Supplementary Fig. S3C), indicating that IL-22 dampened production of multi-functional T cells in acute viral infection. Consequently, over-expression of IL-22 resulted in a delayed viral clearance in tissues (Fig. 6E).

To confirm our findings with IL-22-expressing plasmid, we next injected infected mice with recombinant IL-22 protein (rIL-22). Similarly, we found that CD4^+^ and CD8^+^ T cell numbers in the spleen and liver were reduced by rIL-22 (Fig. 6F). Administration of rIL-22 resulted in decreased numbers of thymocytes and also slightly reduced the counts of single- and double-positive T cells in the thymus (Supplementary Fig. S3D and E). Importantly, rIL-22 inhibited the proportion and number of viral-specific IFN-γ^+^TNF-α^+^ CD4^+^ and CD8^+^ T cells in the spleen and liver (Fig. 6G, Supplementary Fig. S3F). The rIL-22 treatment also resulted in decreased percentages of TNF-α^+^ cells when gated on IFN-γ^+^ T cells (Supplementary Fig. S3F). Accordingly, a higher viral burden in the liver tissues was observed in rIL-22-treated mice (Fig. 6H). Therefore, these data demonstrated that IL-22 possesses impressive features to temper antiviral T cell responses in both lymphoid and non-lymphoid tissues during acute viral infection.

### IL-22 negatively impacts T cell responses in persistent viral infection

**Figure 7:**
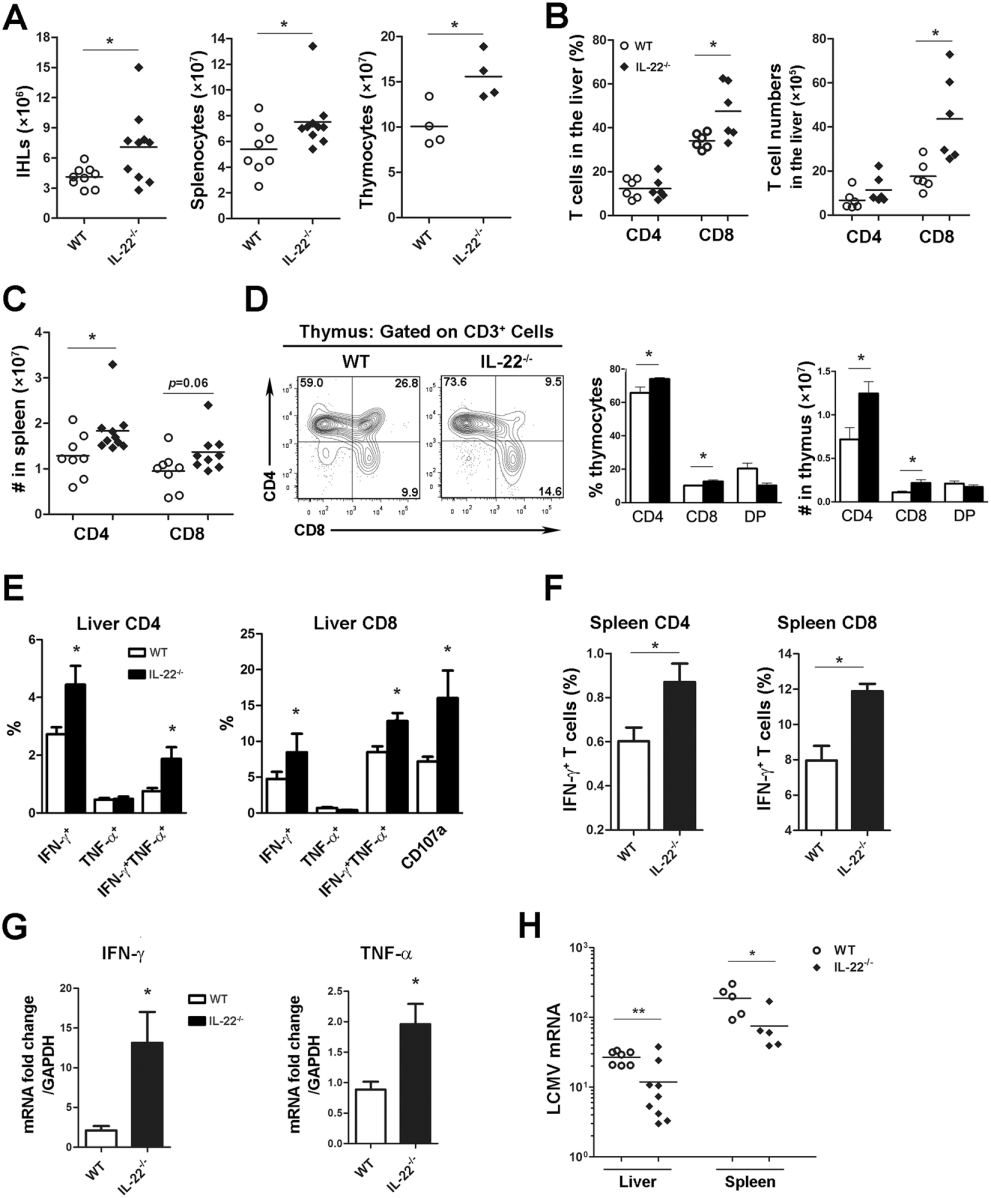
IL-22 deficiency sustains T cell responses in persistent viral infection. WT and *IL*-*22* ^−/−^ mice were infected with Clone 13 (2 × 10^6^ pfu/mouse) for 60 days. (**A**) The total number of leukocytes in the liver, spleen and thymus were counted. (**B**) Percentage and number of CD4^+^ and CD8^+^ T cells in the liver were analyzed with flow cytometry. (**C**) The number of CD4^+^ and CD8^+^ T cells in the spleen was determined. (**D**) Percentage and number of CD4^+^, CD8^+^ and double-positive T cells in the thymus were analyzed with flow cytometry. (**E** and **F**) Leukocytes were isolated and incubated with LCMV peptide GP 61 or GP 33 for 5 h. Cytokine production in viral-specific T cells in the liver (**E**) and spleen (**F**) were measured by flow cytometry. *IFN*-γ and *TNF*-α gene expression in the liver (**G**) and LCMV mRNA in the liver and spleen tissues (**H**) were detected by qPCR. Data are representative of at least two independent experiments. The results are expressed as the mean ± SEM (*n* = 6–10). *p < 0.05.

During chronic viral infection, effector CD8^+^ T cells are eventually exhausted, exhibit poor antiviral function, and lose memory potential^30^. Here, we found that IL-22 deficiency resulted in an increased frequency of memory CD8^+^ T cells in the spleen and liver at 30 dpi (Supplementary Fig. S4A). In this case, IL-22 deficiency resulted in slightly lower levels of AST and viral burden in the liver and lung tissues at 30 dpi (Supplementary Fig. S4B and C). An absence of IL-22 augmented the number of lymphocytes in the liver, spleen and thymus at 60 dpi (Fig. 7A). A lack of IL-22 resulted in an increased percentage and number of CD8^+^ T cells in the liver (Fig. 7B), a greater frequency of CD4^+^ and CD8^+^ T cells in the thymus, and elevated numbers of CD4^+^ and CD8^+^ T cells in the spleen and thymus (Fig. 7C and D).

Importantly, both CD4^+^ and CD8^+^ T cells in the liver from *IL*-*22* ^−/−^ mice exerted higher viral-specific IFN-γ and TNF-α cytokine production and degranulation activity (Fig. 7E). Intensive T cell function was also observed in the spleen of *IL*-*22* ^−/−^ mice (Fig. 7F). Higher levels of IFN-γ and TNF-α gene expression were detectable in the liver tissues of *IL*-*22* ^−/−^ mice compared with those seen in WT mice (Fig. 7G). Consequently, *IL*-*22* ^−/−^ mice exhibited a lower viral load in the liver and spleen compared with that in WT mice at 60 dpi (Fig. 7H). Thus, these data suggest that IL-22 is important for controlling antiviral T cell responses in persistent viral infections. In addition, IL-22 deficiency did not alter the percentage of PD-1-expressing CD4^+^ and CD8^+^ T cells, and slightly increased the proportion of memory CD8^+^ T cells in the spleen at 60 dpi (Supplementary Fig. S4D and E). These data suggest that, in the context of chronic infection, T cells with improved function and memory potential in *IL*-*22* ^−/−^ mice may not be exhausted more quickly than those in WT mice.

## Discussion

Although IL-22 has been identified as a protective cytokine by its direct inhibition of cellular apoptosis and because it favors tissue regeneration in the liver and gut, the actions of IL-22 vary among different organs and disease status^1, 31^. Until now, the role of IL-22 has not been well-defined in viral immune pathogenesis. In this study, in response to viral infection, IL-22 production was elicited in the liver, as well as in lymphoid organs such as the thymus and spleen. When compared with the thymus and spleen, the liver has been proven to be the major organ that produces IL-22 following viral exposure. More IL-22-producing γδ T cells may be responsible for the liver tissues’ ability to produce higher levels of IL-22 compared to those from the thymus and spleen. In contrast to the intestinal IL-22^+^ ILCs^32^, hepatic ILCs predominantly produce IL-17 rather than IL-22 in LCMV infection, implying heterogeneity in the origin and functions of ILCs among various organs. Interestingly, although IL-22 was detected in tissues, it was below the detection limit in the sera of both naïve and infected mice (Fig. 1A). This result suggests that IL-22 is mainly produced by tissue-resident cells, such as γδ T cells in the liver and ILCs in the gut, which play a unique role in local microenvironments.

The molecular mechanism of IL-22 production in γδ T cells during viral infection remains elusive. In the current study, we found that IL-22 production in γδ T cells was regulated through an IL-23/PI3K/mTORC1 axis (Fig. 2). We demonstrated that IL-23 can activate the PI3K/mTORC1 signaling pathway, which prominently regulates IL-22 production in γδ T cells both *in vitro* and *in vivo*. In addition to γδ T cells, hepatic DNT cells have also been proven to be another source of IL-22 during viral infection. They show a pattern similar to that of γδ T cells in cytokine production and underlying regulatory mechanisms (Supplementary Fig. S5). These results corroborate findings in neutrophils in a murine colitis model^33^, and highlight the function of the PI3K/mTORC1 signaling pathway in mediating IL-22 production in innate cells. Importantly, we found, differing from previous studies which conclude that AhR is a positive inducer of IL-22 in the gut immunity^29^, that AhR signaling principally controlled IL-22 production in the absence of IL-23, but was dispensable for regulating IL-22 production in the presence of IL-23 during viral infection (Fig. 3). This finding suggests to us that due to the disease status^34^, AhR and IL-23 may regulate IL-22 production in different ways. In viral infection, pro-inflammatory cytokines (*e*.*g*. IL-23) largely promotes IL-22 production from γδ T cells, which probably overcome the effects of AhR signaling on IL-22 production. Thus, innate IL-22 expression is more likely dependent on an inflammatory microenvironment. Further study will be needed to assess whether this regulation is specific for γδ T cells or if it also occurs in other IL-22-producing cells, including Th22, NK22, and ILC3, and to clarify this mechanism in different disease models.

Adaptive T cells, both CD4^+^ and CD8^+^, play protective roles in viral infections in animal models^35, 36^. Our data show that IL-22 exhibits the feature of impeding antiviral T cell responses in the liver, as well as in the spleen and thymus during both acute and chronic viral infections. Compared with the liver, the structure and function of the spleen and thymus are greatly affected by IL-22 (Figs 4 to 6). These results imply that, in viral infection, IL-22 may prefer to act on the lymphoid pools and profoundly change T cell functions, and the T cell events in the parenchymal tissues such as the liver may possibly be a consequence of this profile of IL-22.

IL-22Rα1 chain is reported to be restricted to cells of non-hematopoietic origin such as hepatocytes, and types of epithelial cells^1^. Interestingly, IL-22R expression has been validated on thymic epithelial cells in a model of irradiation thymic injury^37^, and on stromal cells of lymphoid nodes in an animal model of collagen-induced arthritis^38^. In viral infection, our data also showed that IL-22R was expressed on thymic and splenic stromal cells (Supplementary Fig. S2A). The IL-22/IL-22R interaction has been reported to drive endogenous thymic regeneration following irradiation insult^37^ and to facilitate germinal center reaction in the lymph nodes in collagen-induced arthritis^38^. However, in contrast to its protective role in lymphoid tissues, administration of IL-22 also has caused thymic atrophy in an adenovirus-mediated delivery system^18^. In agreement with reports of IL-22’s damaging effects in lymphoid tissues observed in adenovirus delivery, our data show that excessive IL-22 prominently induces thymic and splenic atrophy in acute viral infection, leading to the inhibition of T cell development and detriment to immune functions. Although the spleen and thymus shrank in control plasmid group compared with those in steady states, IL-22 plasmid caused significant atrophy in these tissues compared with control plasmid (Fig. 6A and B). Moreover, rIL-22 treatment resulted in consistent effects on thymic T cell output, indicating that such suppressive effects are likely to be induced by IL-22 rather than by toxicity of the IL-22 plasmid. However, it remains possible that the inhibitory action of IL-22 may result from its direct cytotoxicity. Given that lymphoid stroma has been demonstrated to inhibit activated T cell proliferation in lymphoid organs^39^, we speculate that IL-22 induces inhibitory factors in IL-22R^+^ lymphoid stromal cells possibly through the STAT pathway, leading to the inhibition of T cell proliferation and function in lymphoid organs (Supplementary Fig. S6). In addition, in the model of irradiation injuries or collagen-induced arthritis, the epithelial cells are greatly and mainly damaged in the thymus^37, 38^, which is likely to highlight the protective role of IL-22 in these cells. However, in viral infections, which involve more remarkable T cell activation in the lymphoid organs, the IL-22/IL-22R axis may principally play a regulatory role in T cell responses in lymphoid organs^18^. Therefore, IL-22 appears to play a dual role in lymphoid tissues according to the tissue microenvironment. Further study is needed to elucidate the checkpoint mechanism for the outcome of IL-22 in certain contexts.

Previous studies of HBV or HCV infection *in vitro*, or in HBV transgenic mice, have reported that IL-22 failed to affect viral replication since it did not induce expression of IFN-stimulated genes^31, 40, 41^. Our data, however, revealed that IL-22 suppresses antiviral effects in T cells and subsequently impedes viral elimination during acute and persistent viral infections (Figs 5 to 7). Although IL-22 does not induce IFN-stimulated genes against viral infection within an *in vitro* cell culture system, it is capable of regulating antiviral T cell responses in the host. In the HBV transgenic mouse model, HBsAg-immunized splenocytes were transferred to HBV.CB6F1 transgenic mice and exerted antiviral activities in the recipient mice^16^. However, IL-22 neutralization does not directly modulate the function of these transferred cells, since they do not express IL-22 receptor. Therefore, IL-22 neutralization fails to control viral replication in this HBV transgenic model. Since data from our virus-infection model show that IL-22 may influence the lymphoid pools via IL-22R-expressing stromal or epithelial cells, the effects of IL-22 on viral replication may depend on an inflammatory immune environment. In addition, persistent infections with HIV, HBV, HCV and LCMV are often associated with T cell dysfunction or exhaustion^30, 42–46^. Our data show that IL-22 deficiency exhibits an unrelenting effect on promoting antiviral T cell responses during persistent viral infection (Fig. 7 and Supplementary Fig. S4). An enhanced ability to produce cytokines from viral-specific T cells in *IL*-*22* ^−/−^ mice is accompanied by improved T cell memory potential at 30 and 60 dpi, without elevated PD-1 expression (Supplementary Fig. S4). Additionally, viruses in the spleen and liver tissues of *IL*-*22* ^−/−^ mice at 60 dpi were almost cleared (Fig. 7H). We therefore speculate that an elevated T cell response in *IL*-*22* ^−/−^ mice may not lead to overt T cell exhaustion and/or viral persistence.

A previous study reported that IL-7 promoted IL-22 production and enhanced thymic output in LCMV infection^47^. In addition to the ability to produce IL-22, IL-7 has been shown to directly regulate thymocyte development, division, and survival through the IL-7 receptor and the activation of its downstream PI3K pathway^48–50^. Our data show that IL-22 suppresses effector T cell output from the thymus (Figs 4 and 6, Supplementary Fig. S3), which is consistent with other reports that IL-22 induces thymic atrophy^18^. Thus, the effects of IL-7 on thymic output may be due to its direct function on thymocytes rather than to the influence of IL-22. In addition, it has been reported that IL-22 neutralization for 1 day did not alter AST levels in Clone 13 infection at 22 dpi^47^. This finding is consistent with our observation that IL-22 deficiency only slightly decreased AST or ALT levels at 7 and 30 dpi, respectively (Fig. 5C and Supplementary Fig. 4B). Interestingly, the protective effect of IL-22 on hepatocytes is dependent on IL-7 treatment, which significantly increases production of multiple inflammatory cytokines, including IL-6, IL-17, IL-12, IL-1, and IFN-γ^47^. These findings suggest to us that IL-22 mainly plays a protective role against IL-7-induced inflammation.

In summary, our data demonstrate that viral infection triggers a γδ T cell-derived IL-22 production, which is mediated by the IL-23/PI3K/mTORC1 signaling pathway. IL-22 is critical for controlling antiviral T cell responses both in lymphoid and non-lymphoid tissues during acute and persistent viral infections. These findings enhance our understanding of the actions of IL-22 in viral infection and imply potential immunotherapy for human viral infectious diseases.

## Methods

### Antibodies and reagents

All fluorochrome-labeled antibodies were purchased from eBioscience (San Diego, CA), BD Pharmingen (San Diego, CA), BD Biosciences (San Jose, CA) and Biolegend (San Diego, CA). The following antibodies were purchased from eBioscience: PE-anti-mIL-22 (1H8PWSR), PE-Cy7-anti-mIL-17A (eBio17B7), APC-anti-mIFN-γ (XMG1.2), PerCP-eFluor 710-anti-mTNF-α (MP6-XT22), PE-Cy7-anti-mCD3 (17A2), PerCP-eFluor 710-anti-mCD3 (17A2), Pacific blue-anti-mCD4 (GK1.5), APC-eFlour 780-anti-mCD8 (53–6.7), APC-anti-mTCRγδ (eBioGL3), PE-anti-mCD44 (IM7), PE-anti-mCXCR3 (CXCR3-173), PE-anti-mKi67 (SolA15), and eFluor 506-conjugated fixable viability dye. Purified anti-mCD16/32 (2.4G2) was purchased from BD Pharmingen. FITC anti-mTCR Vγ1.1/Cr4 (2.11) was purchased from Biolegend. FITC anti-mTCR Vγ4 (UC3-10A6) was obtained from BD Biosciences. The mAb for Vγ6 (17D1) was a gift from Dr. Robert Tigelaar (Yale School of Medicine, CT). PE-anti-mPhospho-S6 ribosomal protein (Ser235/236) (D57.2.2E) was purchased from Cell Signaling Technology (Danvers, MA). Recombinant murine IL-23 and IL-22 proteins were purchased from Peprotech (Rocky Hill, NJ). The mTORC1 inhibitor (rapamycin) and PI3K inhibitor (Ly294002) were purchased from Calbiochem (San Diego, CA). AhR inhibitor (AhRi, CH-223191) was purchased from Sigma-Aldrich (St. Louis, MO).

### Animals and viruses

C57BL/6 (B6) mice (6-8-weeks-old) were purchased from the Jackson Laboratory (Bar Harbor, ME). *IL*-*22* ^−/−^ mice on the B6 background were kindly provided by Dr. Wenjun Ouyang of Amegen (Thousand Oaks, CA). *AhR* ^−/−^ mice (C57BL/6-*Ahr* ^tm1.2Arte^) were obtained from Taconic (Hudson, NY). *TSC1* ^fl/fl^ mice (*TSC1* ^tm1Djk/J^) were from The Jackson Laboratory. LCMV strains Clone 13 and Arm were gifts from Dr. Maria Salvato (University of Maryland, MD). To induce acute hepatitis, we *i*.*v*. injected mice with Clone 13 (2 × 10^6^ pfu/mouse) or Arm (2 × 10^5^ pfu/mouse) for 7 days. To induce a persistent viral infection, we *i*.*v*. injected mice with Clone 13 (2 × 10^6^ pfu/mouse) for 30–60 days. All animal experiments were performed according to the National Institutes of Health Guide for Care and Use of Experimental Animals. All procedures were reviewed and approved by the Institutional Animal Care and Use Committee at the University of Texas Medical Branch, Galveston.

### Propagation and quantitation of viruses

The LCMV stocks were prepared and titrated according to procedures in a previous report^51^. Briefly, viruses were incubated with baby hamster kidney cells (BHK, a gift from Dr. Lihong Zhang, University of Texas Medical Branch at Galveston, TX) for 72 h. Then the culture medium was centrifuged for 10 min at 350 × g, 4 °C, and the supernatant was stored at −70 °C. For quantitation of the virus, Vero cells (a gift from Dr. Lihong Zhang, University of Texas Medical Branch at Galveston) were cultured with a series of 10-fold virus dilutions for 60 min. An overlay containing 0.8% methylcellulose (Sigma-Aldrich) was added into the wells. After 4 days’ culture, immunocytochemistry was performed by using mouse anti-LCMV polyclonal antibody (gift from Dr. Robert Tesh, University of Texas Medical Branch, Galveston, TX)^52^ and the number of positive clusters was counted, followed by the calculation of viral titers.

### Hydrodynamic IL-22 delivery

B6 mice were infected with LCMV Arm (2 × 10^5^ pfu/mouse) at day 0, and then hydrodynamically injected with IL-22-expressing plasmid (pRK5-mIL-22, pIL-22, 10 µg/mouse) or control vector (pRK5, 10 µg/mouse) at 3 and 5 dpi, according to procedures in a previous report^32^. The pRK5-mIL-22 and pRK5 plasmids were gifts from Dr. Yangxin Fu (UT Southwestern Medical Center, Dallas, TX).

### TSC-1 knockout mice model

According to previous methods^25–27^, *TSC*-*1* ^fl/fl^ mice were *i*.*v*. injected with Cre-expressing vector (AAV5/Cre, 1 × 10^7^ pfu/mouse) or control vector (AAV5, 1 × 10^7^ pfu/mouse). Three weeks later these mice were challenged with Clone 13 (2 × 10^6^ pfu/mouse). AAV5/Cre and AAV5 vectors were purchased from the Gene Therapy Center Vector Core, the University of North Carolina at Chapel Hill (Chapel Hill, NC).

### Isolation of splenocytes, thymocytes and intrahepatic lymphocytes

The spleen and thymus were mechanically dissociated. The supernatant was collected and incubated with red blood cell lysis buffer (BD Biosciences) to remove red blood cells. IHLs were isolated according to our previous method^52^. Briefly, liver tissue was pressed and digested with collagenase IV (0.05%, Roche Applied Science, Indianapolis, IN) at 37 °C for 30 min. After digestion, cell suspensions were passed through 70-μm nylon cell strainers to yield single-cell suspensions. Cells were purified by centrifugation (400 × g) at room temperature for 30 min over a 30%/70% discontinuous Percoll gradient (Sigma-Aldrich). Then cells were collected from the interphase, thoroughly washed, and re-suspended in complete RPMI-1640 medium containing 10% FBS. The relative percentages of lymphocyte subpopulations were measured by flow cytometry, and their absolute numbers were calculated according to their percentages and the total numbers.

### Flow cytometry

Intracellular staining was performed with flow cytometry according to our previously reported methods^53^. Briefly, for detecting IFN-γ and TNF-α, cells were incubated 5 h with LCMV GP61 peptide (5 μg/ml, AnaSpec, Fremont, CA) or GP33 peptide (5 μg/ml, AnaSpec) for viral-specific CD4^+^ or CD8^+^ T cells, respectively. For IL-22 and IL-17A detection, cells were cultured with or without rIL-23 (20 ng/ml) overnight. Brefeldin A solution (eBioscience) was added at the last 4h of culture. After incubation, cells were collected, stained with fixable viability dye, blocked with Fc receptors blocker (CD16/32), and stained for specific surface molecules. After surface staining, cells were fixed, permeabilized, and stained for intracellular cytokines by using a fixation/permeabilization kit (eBioscience). For phospho-S6 detection, cells were cultured in 96-well plates (2 × 10^6^ cells in 50 μl RPMI-1640 medium with 2% FBS) for 2 h, and then stimulated with rIL-23 (20 ng/ml) for 0, 15, 30, and 60 min, respectively. These cells were collected and fixed with phosflow lyse/fix buffer (BD Biosciences) for 15 min at 37 °C, followed by spinning down and adding phosflow perm buffer III (BD Biosciences) on ice for 30 min. After washing, cells were stained for surface markers and phospho-S6. All samples were processed on an LSRII FACS Fortessa (Becton Dickinson, San Jose, CA) and analyzed by using FlowJo software (TreeStar, Ashland, OR).

### Real-time PCR

Frozen tissues were used to extract total RNA, for which we used an RNeasy Mini kit (Qiagen, Valencia, CA), followed by digestion with DNase I (Ambion, Thermo Scientific, Hudson, NH). The cDNA was synthesized by using an iScript Reverse Transcription Kit (Bio-Rad, Hercules, CA). Then cDNA was amplified in a 10-µl reaction mixture containing 5 µl of iTaq SYBR Green Supermix (Bio-Rad) and 0.9 µM each of gene-specific forward and reverse primers. The PCR assays were denatured for 30 s at 95 °C, followed by 40 cycles of 15 s at 95 °C, and 60 s at 60 °C, by utilizing the CFX96 Touch real-time PCR detection system (Bio-Rad). Relative quantitation of mRNA expression was calculated, by using the 2^−ΔΔCt^ method, as the fold increase in expression. The sequences of the forward and reverse gene-specific primers used are listed in Supplementary Table S1.

### ELISA assays

For detecting IL-22 and IL-17 levels in the supernatants of cell cultures, cells were incubated for 3 days, and then the supernatants were collected, followed by an ELISA assay with commercial kits (eBioscience). For IL-22 detection in tissues, briefly, proteins were extracted from tissues by homogenization on ice in the RIPA Buffer (Cell Signaling Technology) with a protease inhibitor cocktail (Sigma-Aldrich). After centrifugation at 20,000 × g for 20 min, the supernatants were collected, and protein concentrations measured with a protein assay kit (Bio-Rad). Equal amounts of proteins (100 μg) were loaded for ELISA assays by using commercial kits (eBioscience).

### Statistical analysis

The difference between two different groups was determined by using Student’s t test. One-way ANOVA was used for multiple group comparisons (GraphPad Prism 5). P values < 0.05 were considered significant (*), <0.01 as highly significant (**), and <0.001 as remarkably significant (***).

## Electronic supplementary material

Supplementary file
