# Supplementary material for: A tightly regulated IL-22 response maintains immune functions and homeostasis in systemic viral infection
Source: Sci Rep. 2017 Jun 20;7:3857. doi: 10.1038/s41598-017-04260-0 (PMC5478593; doi:10.1038/s41598-017-04260-0)
Supplement: Supplementary file 1 — Supplementary file [file 41598_2017_4260_MOESM1_ESM.pdf]

Supplementary Information

A tightly regulated IL-22 response maintains immune functions and homeostasis in systemic viral infection

Panpan Yi, Yuejin Liang, Denley Ming Kee Yuan, Zuliang Jie, Zakari Kwota, Yan Chen, Yingzi Cong, Xuegong Fan, Jiaren Sun

Supplementary Figure S1: related to Figure 1.

Supplementary Figure S2: related to Figure 5.

Supplementary Figure S3: related to Figure 6.

Supplementary Figure S4: related to Figure 7.

Supplementary Figure S5 and S6: related to Discussion.

Supplementary methods

H&E and histological scores. Liver specimens were fixed in 10% buffered formalin. Paraffin-embedded sections were stained with H&E for histological evaluation by using a modified Ishak grading system[1] (Supplementary Table S2). Briefly, the liver damage degree was evaluated from four separate aspects: periportal necrosis (Score 0-4), portal inflammation (Score 0-4), focal spotty necrosis, or inflammation (Score 0-4).

Isolation of thymic and splenic stromal cells. B6 mice were infected with LCMV Armstrong (2×10^5 pfu/mouse) for 7 days, and then the thymic and splenic stromal cells were isolated according to previously reported methods[2-4]. Briefly, thymic lobes and spleen were minced with scissors respectively. The fragments were dissociated in RPMI-1640 medium containing 2% FBS to release thymocytes or splenocytes, and then settled on ice. The supernatant was discarded and replaced with fresh medium until the supernatant became clear. The fragments were incubated with 1 ml digestion buffer (RPMI-1640 + 2% FBS + 0.4 mg/ml collagenase/dispase (Roche, Indianapolis, IN) + 25 μg/ml Dnase I (Roche) for 30 min at 37°C. Undigested fragments were settled on ice. The supernatant was collected and stored on ice. The remaining fragments were digested a second time in fresh digestion medium. The supernatant was collected and then incubated with stop solution (485 μl RPMI-1640 + 2% FBS + 15 μl EDTA (500mM)) for 5 min at 37°C. The cells were washed and spun down with fresh RPMI-1640 medium. Cells were stained with antibodies (CD45, MHCII,

ebioscience) and detected by flow cytometry.

T cell apoptosis analysis. B6 mice were infected with LCMV Armstrong (2×10^5 pfu/mouse) for 7 days, and then thymocytes and splenocytes were isolated. Cell apoptosis were detected with commercial kits (BD Pharmingen, San Diego, CA). Briefly, cells were washed twice with cold PBS and then resuspended in 1X binding buffer at a concentration of 1×10^6 cells/ml. 100 µl of the solution (1×10^5 cells) were transferred to a new tube and incubated with 5 µl of Annexin V, 5 µl PI and antibodies (CD3, CD4, CD8, ebioscience) for 15 min at RT in the dark. Then 400 µl of 1X binding buffer was added to each tube and samples were analyzed with flow cytometry.

Supplementary tables

Table S1. Primers for qPCR

Gene Name	Primer Sequence
GAPDH	Forward 5'-TGGAAAGCTGTGGCGTGAT-3'<br>Reverse 5'-TGCTTCACCACCTTCTTGAT-3'
IFN-γ	Forward 5'-ATGAACGCTACACACTGCATC-3'<br>Reverse 5'-CCATCCTTTTGCCAGTTCCTC-3'
TNF-α	Forward 5'-ATAGCTCCCAGAAAAGCAAGC-3'<br>Reverse 5'-CACCCCGAAGTTCAGTAGACA-3'
LCMV-GP	Forward 5'- CATTCACCTGGA CTTTGTCAGACTC-3'<br>Reverse 5'- GCAACTGCT GTGTTCCCGAAAC-3'

Table S2. Modified Ishak grade system

Periportal or periseptal interface hepatitis (piecemeal necrosis)	
0	None
1	Mild (focal, few portal area)
2	Mild/moderate (focal, most portal area)
3	Moderate (continuous around < 50% of tracts or septa)
4	Severe (continuous around > 50% of tracts or septa)
Focal (spotty) necrosis, apoptosis, and focal inflammation	
0	None
1	One focus 10X objective
2	Two to four foci per 10X objective
3	Five to ten foci per 10X objective
4	More than 10 foci per 10X objective
Portal inflammation	
0	None
1-2	Mild
2-3	Moderate (some or all portal areas)
3-4	Moderate/marked (all portal areas)

Supplementary Figure S1

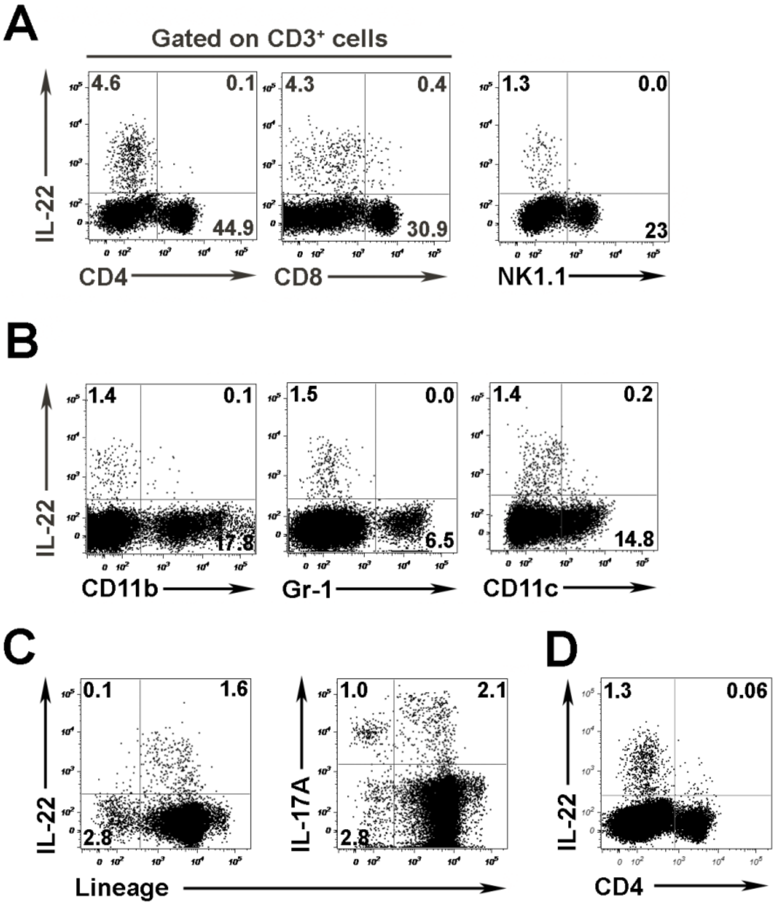

Figure S1. IL-22 expresses in certain leukocytic subtypes in the early stage of viral infection. C57BL/6 (B6) mice were *i.v.* injected with LCMV Clone 13 (2×10^6 pfu/mouse) and then sacrificed at 3 dpi. (**A**) Representative plots for the percentage of IL-22-producing CD4$^+$, CD8$^+$ T cells, and NK cells in the liver. (**B**) IL-22 expression in hepatic myeloid cells was examined by flow cytometry. (**C**) Representative plots of IL-22 and IL-17A expression in hepatic ILCs. Lineage antibodies: CD3, CD4, CD8, CD19, CD11b, CD11c, NK1.1, Gr-1, CD45R (B220), FcεR1. (**D**) Representative plot of IL-22 expression in hepatic total CD4 cells. Data are representative of at least three independent experiments.

Supplementary Figure S2

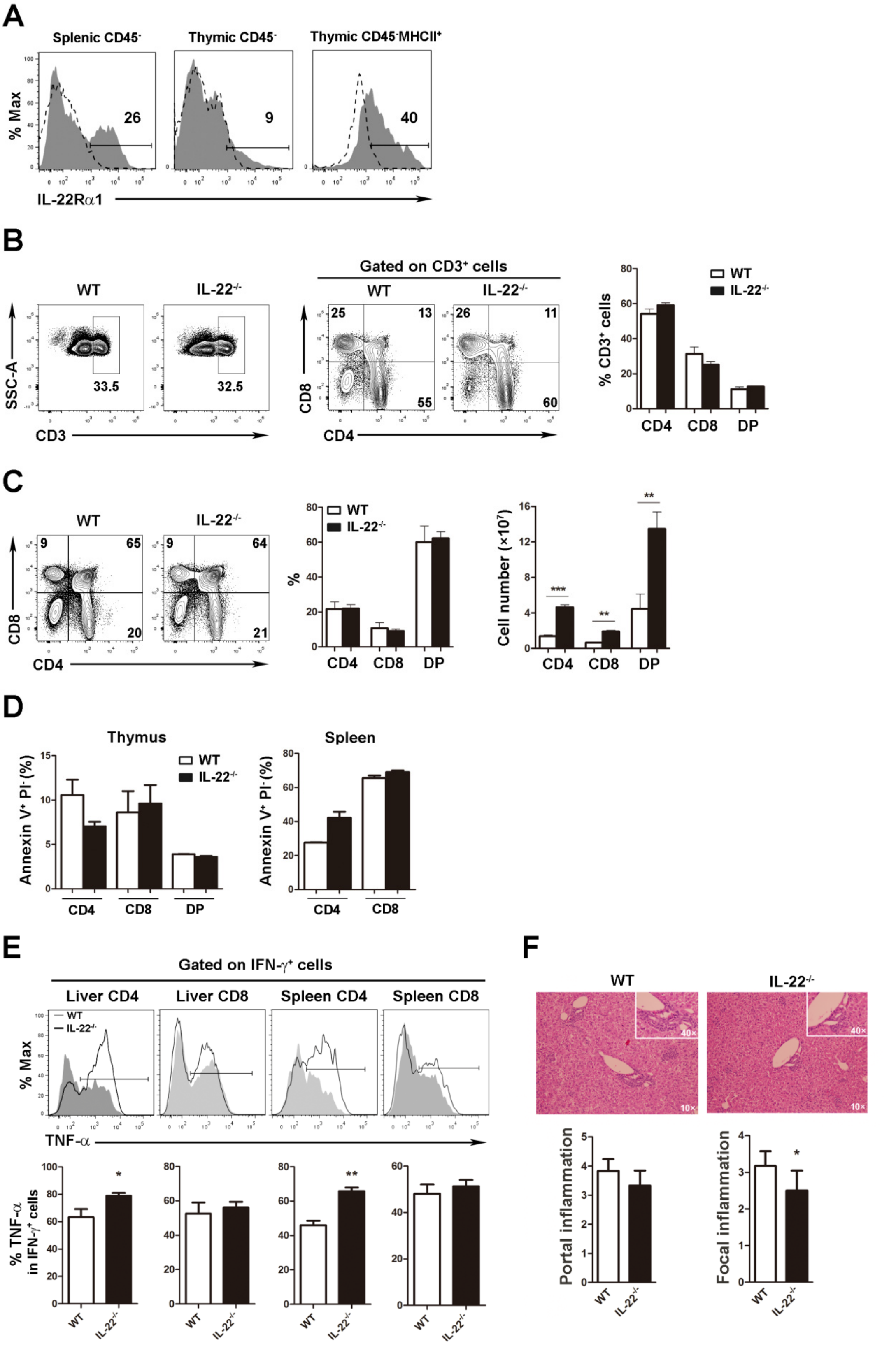

Figure S2. Effects of IL-22/IL-22R on T cell response during acute viral infection.

WT and *IL-22*$^{-/-}$ mice were infected with LCMV Armstrong (2×10^{5} pfu/mouse) for 7 days. (**A**) IL-22Rα expression was detected in isolated thymic and splenic stromal cells with flow cytometry. Splenic stromal cells (CD45^{-}); Thymic non-epithelial stromal cells (CD45^{-}); Thymic epithelial cells (CD45^{-}MHCII^{+}). (**B**) The frequencies of thymic CD4^{+}, CD8^{+}, and DP T cells were determined when gated on CD3^{+} cells. (**C**) The frequencies and counts of single-positive, and DP cells in the thymus were analyzed when gated on total thymocytes. (**D**) The proportion of apoptotic T cells (Annexin V^{+} PI^{-}) in the thymus and spleen was examined by flow cytometry. (**E**) The percentage of TNF-α^{+} cells when gated on IFN-γ^{+} T cells of the infected WT and IL-22$^{-/-}$ mice were examined with flow cytometry. (**F**) Liver histological inflammation scores were evaluated by the modified Ishak grading system (Supplementary Table S2). Data are representative of three independent experiments. The results are expressed as the mean ± SEM (*n* = 3-5 per group). *, p<0.05; **, p<0.01; ***, p<0.001.

Supplementary Figure S3

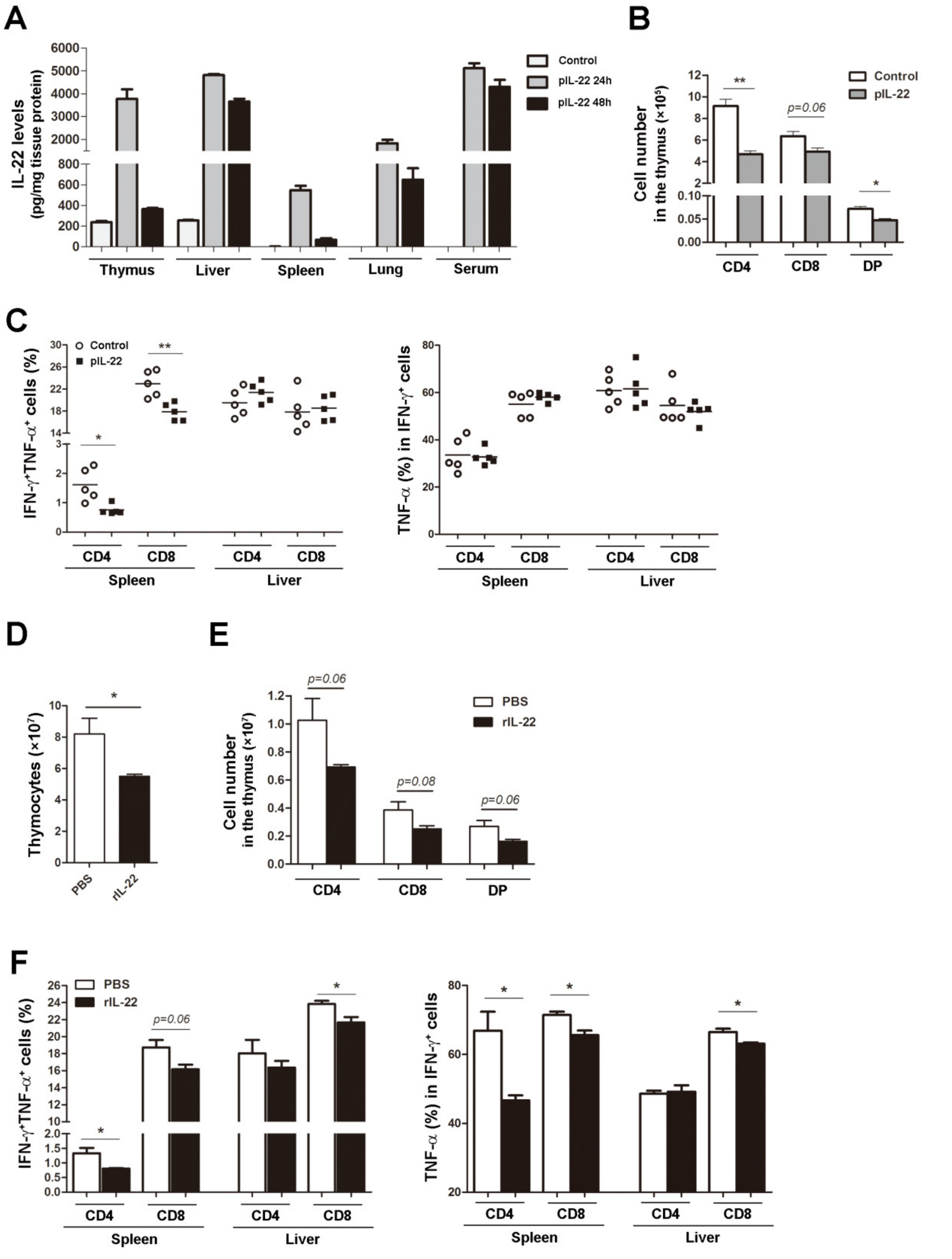

Figure S3. Over-expression of IL-22 tempers T cell responses in acute viral infection. (**A**) B6 mice were infected with LCMV Armstrong (2×10^5 pfu/mouse) and hydrodynamically injected with pRK5-mIL-22 (pIL-22, 10 μg/mouse) or pRK5

(control, 10 μg/mouse). IL-22 levels in thymus, liver, spleen, lung, and serum were tested with ELISA at 24 h and 48 h. (**B** and **C**) B6 mice were infected with LCMV Armstrong (2×10^5 pfu/mouse) at day 0, and then hydrodynamically injected with pRK5-mIL-22 (pIL-22) or pRK5 (control) at 3 and 5 dpi. Mice were sacrificed at 7 dpi. T cell numbers in the thymus (B) and the proportions of IFN-γ^+TNF-α^+, as well as TNF-α^+ population within IFN-γ^+ T cells in the spleen and liver (C) were determined by flow cytometry. (**D** to **F**) B6 mice were infected with Armstrong, and then *i.p.* injected with recombinant IL-22 protein (5 μg/mouse) or PBS at 1, 3 and 5 dpi. At 7 dpi, the counts of thymocytes (D), T cell numbers in the thymus (E), the frequencies of IFN-γ^+TNF-α^+ and TNF-α-expressing IFN-γ^+ T cells in the spleen and liver (F) were analyzed by flow cytometry. Data are representative of two independent experiments. The results are expressed as the mean ± SEM (n = 4-5). *, p<0.05; **, p<0.01.

Supplementary Figure S4

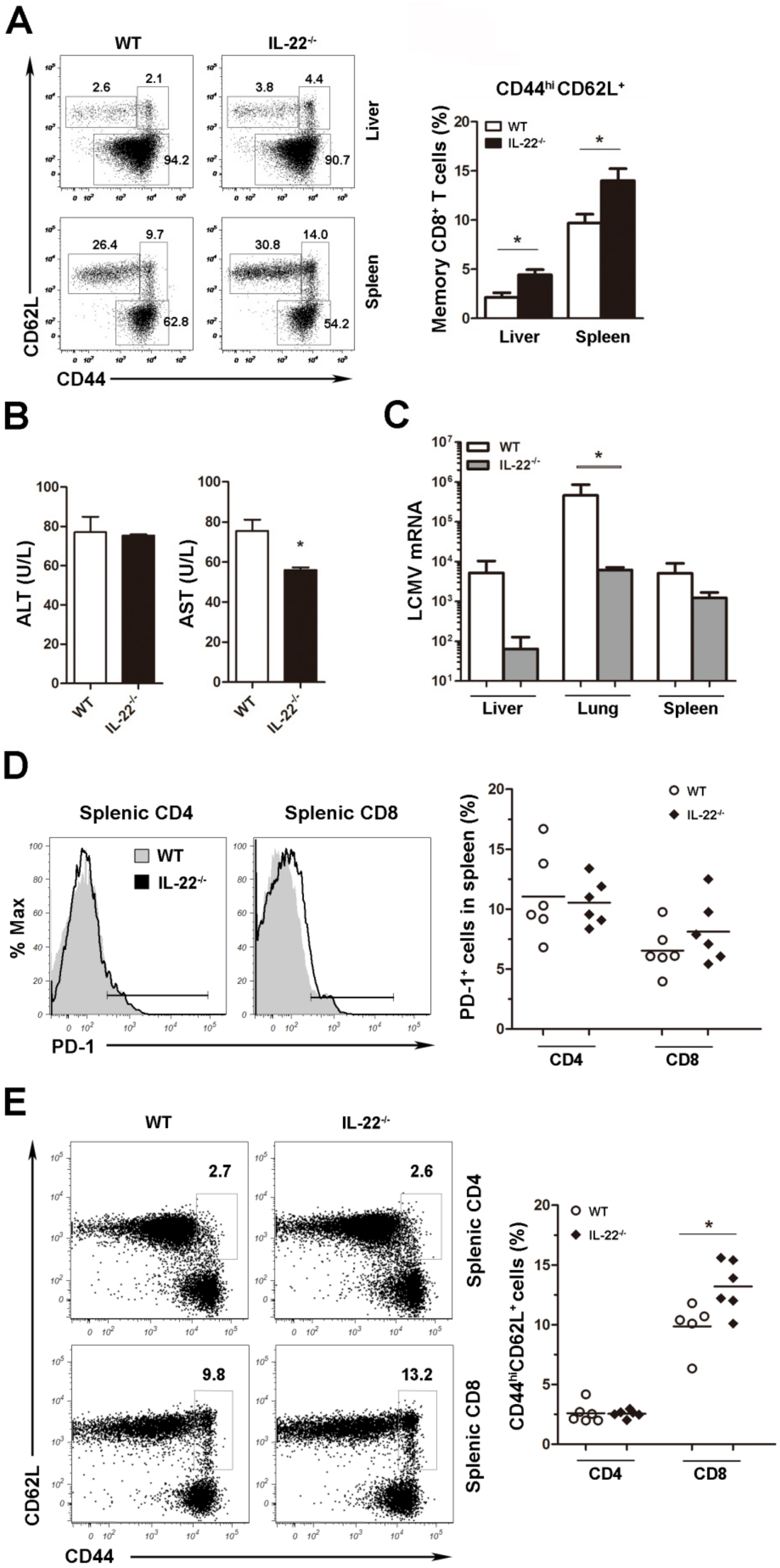

Figure S4. IL-22 deficiency increases frequency of memory CD8^{+} T cells without

elevating PD-1 expression in the spleen during persistent viral infection. (**A** to **C**) WT and *IL-22*$^{-/-}$ mice were infected with Clone 13 (2×10^6 pfu/mouse) for 30 days. (A) Memory $CD8^+$ T cells in the liver and spleen were analyzed by flow cytometry. (B) Serum ALT and AST levels were tested. (C) Expression of viral mRNA in liver, lung and spleen tissues was determined by qPCR. (**D** and **E**) WT and *IL-22*$^{-/-}$ mice were infected with Clone 13 (2×10^6 pfu/mouse) for 60 days. The frequency of PD-1^+ T cells (D) and memory T cells (E) in the spleen were determined by flow cytometry. Data are representative of at least two independent experiments. The results are expressed as the mean ± SEM (n = 4-6 mice per group). *, $p<0.05$.

Supplementary Figure S5

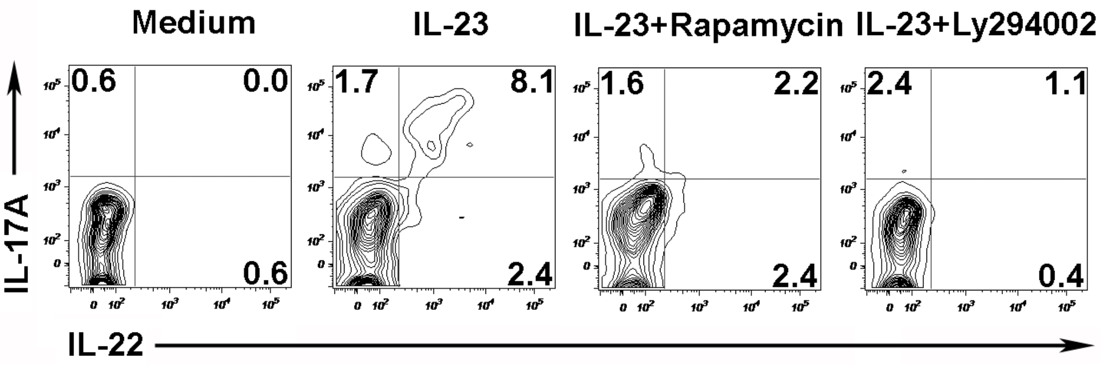

Figure S5. PI3K/mTOR signaling pathway regulates IL-23-induced IL-22/IL-17 expression in DNT cells. IHLs were isolated from Clone 13-infected B6 mice at 3 dpi and then cultured with the indicated conditions overnight. IL-23 (20 ng/ml); Rapamycin (mTOR inhibitor, 25 nM); Ly294002 (PI3K inhibitor, 5 μM). IL-22 and IL-17 expression in DNT cells was analyzed by flow cytometry. Data are representative of three independent experiments.

Supplementary Figure S6

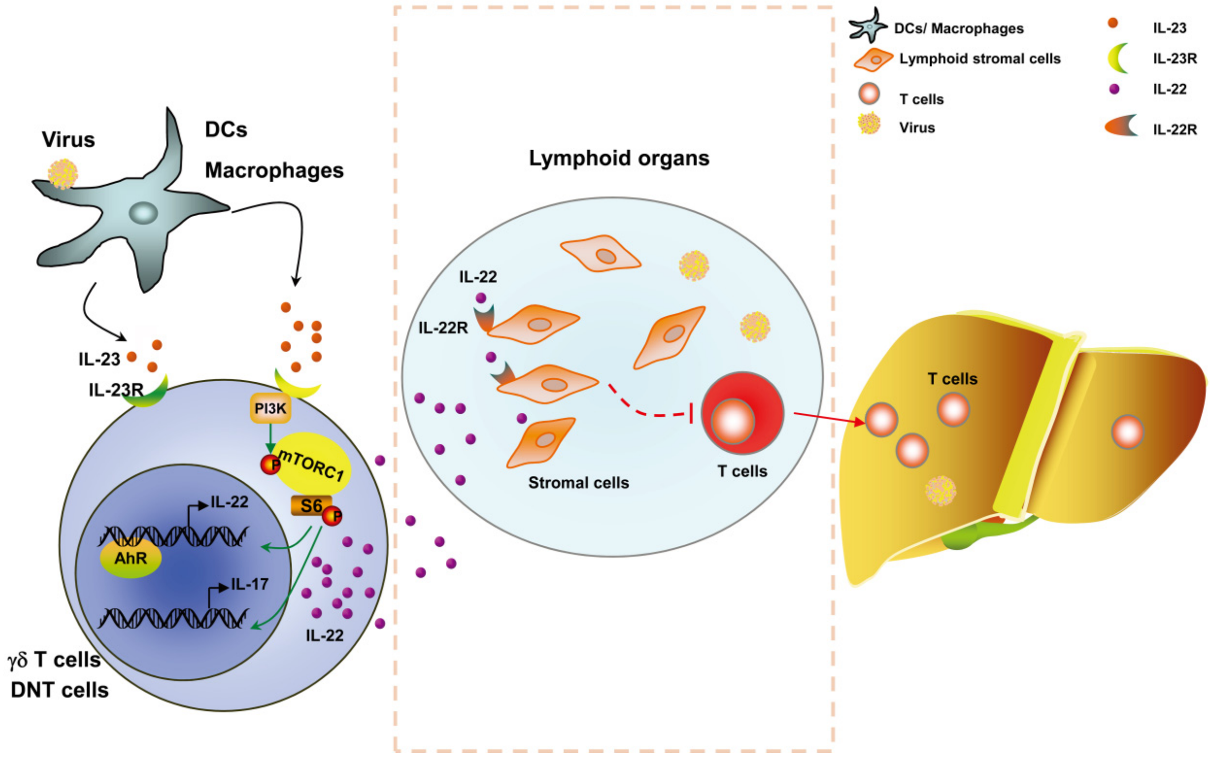

Figure S6. A schematic image of IL-22 in viral infection. In viral infection, IL-23 promotes IL-22 expression in γδ T cells or DNT cells through the PI3K/mTOR signaling pathway. IL-22 subsequently suppresses the infiltration and function of T cells in the liver, potentially by acting on epithelial or stromal cells in lymphoid organs to constrain T cell development and output. This contributes to modulation of T cell responses and maintaining immune homeostasis in viral infection.

References

1 Goodman, Z. D. Grading and staging systems for inflammation and fibrosis in chronic liver diseases. *J Hepatol*. **47,** 598-607 (2007).

2 Rode, I. & Boehm, T. Regenerative capacity of adult cortical thymic epithelial cells. *Proc Natl Acad Sci U S A*. **109,** 3463-3468 (2012).

3 Boehm, T., Scheu, S., Pfeffer, K. & Bleul, C. C. Thymic medullary epithelial cell differentiation, thymocyte emigration, and the control of autoimmunity require lympho-epithelial cross talk via LTbetaR. *J Exp Med*. **198,** 757-769 (2003).

4 Gray, D. H. *et al.* Unbiased analysis, enrichment and purification of thymic stromal cells. *J Immunol Methods*. **329,** 56-66 (2008).
